# ‘It just opens up their world’: autism, empathy, and the therapeutic effects of equine interactions

**DOI:** 10.1080/13648470.2017.1291115

**Published:** 2017-05-17

**Authors:** Roslyn Malcolm, Stefan Ecks, Martyn Pickersgill

**Affiliations:** a Department of Social Anthropology, University of Edinburgh, EdinburghEH8 9LD, United Kingdom; b Department of Anthropology, University of Edinburgh, Edinburgh, United Kingdom; c Usher Institute for Population Health Sciences and Informatics, Edinburgh Medical School, University of Edinburgh, EdinburghEH8 9AG, United Kingdom

**Keywords:** Autism, horse-assisted therapy, efficacy, human–animal relations, intersubjectivity, empathy

## Abstract

Experiences of autism-spectrum disorder are now increasingly studied by social scientists. Human–animal relations have also become a major focus of social inquiry in recent years. Examining horse-assisted therapy for autistic spectrum disorders, this is the first paper that brings these fields together. Drawing on participant observation and interviews at a UK horse therapy Centre, this article examines how staff and the parents of riders account for the successes and limitations of equine therapy. To the respondents, horses ‘open up’ autistic children and make possible interactions that seemed impossible before. Horses were regarded as facilitating the emergence of apparently social behaviours, which included eye contact, pointing, and speech. Three key explanations emerged for therapeutic success: the sensorial, embodied experience of riding the horse; the specific movements and rhythms of the horse; and, the ‘personality’ of the horse. Equine therapy can be regarded as enabling a form of multispecies intersubjectivity, with the resonance between rider and horse seeming to make possible a new attunement between humans. Practices of equine therapy, and perceptions of its efficacy, serve in turn to attune social scientists to a version of empathy constituted through lively and sensorial interactions, as opposed to one that is restricted to particular kinds of humans.

## Introduction

How can horse riding have therapeutic effects on people living on the autism spectrum? We seek to address this question through a study on human–animal relations at a UK equine therapy Centre. Based on participant observation of autistic^1.^ riders and interviews with parents and therapeutic staff, we propose that the efficacy of horse-assisted therapy for autism seems to emerge from a dynamic attunement of riders, horses, parents, staff, and the physical environment of the Centre. We analyse participants’ perspectives on the therapeutic aspects of equine encounters with an emphasis on the effects of therapy and the possible reasons for why it is considered successful. Troubling notions of autism as a strictly individualized and brain-based condition, we show that an ethnographic approach that is sensitive to all the participants' experiences can cast light on therapeutic effects provided by structured yet hard to verbalize human–animal interactions. We reflect on how experiences of horse-based therapy can push us to think differently about dynamics of intersubjectivity that emerge from a crossing of species boundaries.

This study lies at the intersection of two areas of research in the social sciences, one concerned with autism, another concerned with human–animal relations (particularly in terms of human health). With regards to the latter, over the past decade the social sciences, and especially anthropology, have become more interested in human–animal relations than ever before (e.g. Kirksey and Helmreich 2010; Hurn 2012; Hutton 2015; Friedmann et al. 2013; Jadhav and Barua 2012; Jalais 2010; Kelly and Lezaun 2014). Furthermore, anthrozoological research specifically concerned with animal-assisted therapies has begun to flourish. One meta-analysis of animal-assisted therapies (Nimer and Lundahl 2007), for example, underscores the diversity of work in this area. It also attends to the apparent efficacy of particular therapies, such as animal assisted techniques that enhance emotional well-being and lessen autism-spectrum symptoms. Prothmann, Ettrich, and Prothmann (2009) also found that for children with autism, dogs’ communications were more easily accessible than those of humans. Solomon (2012) similarly found that due to easily readable communication of intentions, a dynamic of inter-subjectivity emerged between therapy dogs, autistic children, and other family members, helping to facilitate communication.

In spite of these studies, a focus on horses in the autism therapy context is lacking. We address this limitation within the literature, and in so doing engage with other ethnographies of human–horse relations beyond the realm of health per se (Cassidy 2002; Hurn 2008, 2012; Latimer and Birke 2009). In particular, analytic cues are taken from analyses of the complexity of human–horse interactions (Latimer and Birke 2009; Hurn 2008), and the partial intersubjectivity (Cassidy 2002) that relates to this (which, as Hurn (2012) suggests, requires an acknowledgement of the mindedness of the animal). More generally, conceptions of agency have been a major focus of multispecies ethnographers, with the agentic capacities of humans seen as co-constituted by nonhuman actors. In essence, humans and animals enter into a mutually affecting ‘ontological choreography’ (Haraway 2003, 51). Hence, and in line with previous studies, our analysis of horse-assisted autism therapy tentatively understands agency as performative and co-constitutive across species boundaries (Latimer 2013; Maurstad, Davis and Cowles 2013; Irvine 2014).

As anthropological interest has developed in animal-human interactions, so too has wider social scientific interest in autism (Fein 2015; Fitzgerald 2012; Hollin and Pilnick 2015; Silverman 2012; Solomon 2010). Scholars have often explored autistic sociality (Bagatell 2010; Broderick and Ne'eman 2008; Grinker 2010; Ortega 2009), and consequently rendered problematic an individualised conception of autism that sometimes results from an exclusively neurological focus or understanding of the condition. In particular, some work has interrogated the dualisms that help to configure autism as ‘the alien within the human, the mystical within the rational, the ultimate enigma’ (Murray 2006, 26). Anthropologists, sociologists, and others have commonly charted the emergence of a view of autism as an expression of ‘neurodiversity’, wherein autism is understood as a form of neurological and behavioural difference rather than an all-encompassing disability. This emphasis on neurodiversity, which acknowledges differences without pathologizing all of them, is also popular among autism self-advocates (Broderick and Ne'eman 2008; Hacking 2009a, 2009b).

This paper engages with questions of efficacy regarding equine therapy for autism. In essence, we ask: does this intervention work, and if so, how? In the case of equine therapy, the few studies that have examined efficacy support the therapeutic efficacy of horse riding (see Gabriels et al. 2012). Similarly, popular media reports of autism therapies tend to display their positive, sometimes even miraculous, effects.^2.^ Here, we explore perceptions of the staff and parents involved in a UK equine therapy Centre regarding how this therapeutic modality works. We take seriously the accounts and intuitions of our respondents, yet also heed the ways in which what is deemed to work plays a role in constituting the target of therapy.

## Autistic intersubjectivity

Among the many aspects of autism that could be explored, this paper focuses explicitly on how horse riding is perceived to ‘open up’ autistic people and allow them to become engaged socially with human and nonhuman others in ways that are therapeutic. These moments of ‘opening’ are so remarkable because they defy (even if only to a limited degree) the ‘triad of impairment’ that is seen as typical for autism: deficits in social interaction, deficits in communication, and repetitive patterns of behaviour, interests or activities (Wing and Gould 1979; American Psychiatric Association 2013).

Autism has long been described as a disorder of sociality. In his reclassification of dementia praecox into schizophrenia a century ago, Eugen Bleuler reported an ‘[a]utistic withdrawal’ (Bleuler 1912, 363). Later, Leo Kanner defined ‘infantile autism’ a disorder with ‘withdrawal tendencies’ (Jacobsen 2010, 439). Bettelheim's *The Empty Fortress* (1967) subsequently articulated autism as a highly constraining boundary to the ‘outside’ social world. It assumed those with the condition were without ‘inner’ experience (Hacking 2009a): uncivilised, uncultured, and uncommunicative – as ultimately incapable of intersubjective relations. In the 1980s, the alleged lack of empathy among autistic people became more salient, entering discussions of autistic symptomatology alongside the innovation of the new category of Asperger's Disorder (Rogers et al. 2007). The emergence of autism as a more prevalent psychiatric category in the 1980s was dependent on this view of the (neurological) individual as the locus for sociality (Hollin and Pilnick 2015). Contemporary scientific and public discourse on autism often frames individuals diagnosed with the condition as limited in their capacities for intersubjective relations and empathy (Silverman 2012). In much neuroscientific writing and research on autism, we can see that intersubjectivity has been framed solely as a process of abstract cognitive representation.

Many autistic people find that empathizing with other humans can be very difficult. However, they also suggest that the lack of understanding ‘cuts both ways’ (Shore 2003, 165): non-autistic people struggle as well to understand autistic people. Individuals living with autism also commonly report heightened sensory awareness as one of the most troubling aspects of the condition (Chamak et al. 2008). Sensory idiosyncrasies include sound, vision, taste, smell, proprioception, sensory overload, and synaesthesia. Difficulties in understanding others and heightened sensory awareness are often experienced as two aspects of the same condition. The well-known scientist and autism activist Temple Grandin puts this in striking terms: ‘our bodies cry out for human contact but when contact is made, we withdraw in pain and confusion’ ([1986] 2005, 36). Such sensory differences from the majority are sometimes expressed by autistic people as positioning them ‘out of place’ in a physical and social geography designed by, and for, neurotypical humans (Davidson 2010). The construction and positioning of the built environment - sensory furniture’ (Darius, quoted in Davidson 2010, 306) – helps to produce and increase autistic disability, and a lack of comprehension of (or, indeed, empathy for) these issues is felt to limit understanding between autistic and non-autistic individuals.

## Methods

This paper explores equine therapy in the context of a UK Centre that has been working with autistic children and horses since the late 1960s. The data collected were the result of Malcolm's ‘observant participation’ (Moeran 2009) over three months at the Centre in 2012. Alongside on-going informal interviews, nine semi-structured interviews were carried out with Centre staff and volunteers, and with the teachers and parents of service users with autism-spectrum disorders. All interviews were recorded and transcribed. Pseudonyms were used for all informants, service users, and special educational needs schools participating in the study. Ethics approval was obtained from the University of Edinburgh. This paper focuses on the perspective of service providers and parents/carers of the autistic people who accessed the Centre for equine therapy sessions.^3.^ The research also builds upon Malcolm's experience as volunteer and employee from 2007 to 2012, allowing access to the ‘back stage’ of the organization.

In addition to having been a staff member at the Centre, Malcolm is a longstanding member of the equestrian community. Learning to work with and ride horses is intrinsically embodied. In order to ride effectively, reactions and bodily movements must become subtle and instantaneous: spontaneous reactions must be unlearned in order to engage with new movements informed by the motion and reflexes of the horse, becoming intuitive with time and experience. Malcolm could thus draw on a ‘habitographic’ (Ecks and Kupfer 2015) sensibility that came from years of work with the people described, in the physical environment described here. Her proximity to the field of study was helpful in terms of prior bodily experience and knowledge, and gaining access to the inner workings of the Centre. It provided a number of years of experience to use as a gauge for responses, at a time when informants were not ‘performing’ for the anthropologist as ‘other’ (Ohnuki-Tierney 1984). Her position in the management of the centre rather than as a practitioner of the therapy ensured sufficient ‘distance’ from the process: she did not ‘go native’.

On-going discourse analysis was used to ascertain the most salient features of the data from which to organise the main themes. Once data saturation occurred, the central themes of the data were used to organise the description of data. Discursive and non-discursive accounts can be complementary rather than mutually exclusive (Csordas 2002) and more empirically tenable than mere subjugation of the bodily to the semantic (Jackson, 1989, as quoted in Csordas 2002). In addition to discourse analysis, then, the shared non-discursive embodied experience of equine therapy – an aspect continually highlighted by informants – was explored.

## A typical therapy session

Children mostly reached the Centre in groups on minibuses from special educational needs schools or units within mainstream schools. The group would get off the bus and head into the Centre, catching a glimpse of the row of stables straight ahead behind a wooden entrance gate, the horses’ heads poking out over the doors. Each session was tightly scheduled, both for the benefit of the school timetable and the smooth running of the Centre which ran up to 10 sessions each day. The children would be taken inside to the sand riding arena with its dim lighting and dampened sounds: past the cafe, and straight up a gently sloping walkway to the mounting block (a raised platform built to facilitate riders with physical disabilities).

The young people would be assisted to the front of the mounting area in turn, as their horse was drawn up to stand and wait quietly below. Each horse was chosen specifically after consultation with the teacher or parent of each child. Once everyone was on, stirrups would be altered, girths tightened, and the session would begin. Depending on the instructor, different games and tasks would be played. First was always a good few laps of walk to warm up, and (if possible) trot. At each transition in pace, the child was encouraged by the instructor or helper to communicate verbally with the horse: ‘say walk on’ or ‘say trot on’, and so forth.

Led by a helper (and assisted by side walkers on either side of the horse if not quite yet balanced to ride alone), each child would take a turn to trot from the front to the ride, to the back. The instructor called ‘up, down, up, down’ in time with the rhythm of the horses’ pace in the hope that this would assist the child to rise and fall from the saddle at the appropriate moments. Each task ended with an encouragement from the helper for the child to pat or stroke and thank the horse. Then, there were games of bending between blocks or stretching towards the horses’ ears and tail. After 30 minutes, the group would often ride out from the cool calm of the arena into the bright light of the grounds, making their way to a wooded trail following the course of a river. After a walk around the trail, the group typically returned to the arena to dismount and say ‘thank you’ and ‘goodbye’ to their horses and helpers. Then it was back onto the minibus and back to school.

## Experiencing autism

For Centre staff, autism was a brain disorder of unknown aetiology. They were clear that horse-assisted therapy could not bring a permanent cure: ‘you're never going to change hugely from being autistic’ (Violet). The trainers nevertheless believed firmly that the horse therapy could greatly improve symptoms. Accepting that people's condition could be improved was an important goal for wider public awareness, too.

While Centre staff located autism primarily in an individual's own cognitive system, they also emphasized how the condition was co-constituted by social interactions in which the autistic person is labelled as pathological and treated accordingly. Millie, a teacher of autistic children, pointed out that the symptoms of autism depend on the social interaction: ‘Because there is a lack of communication, they get frustrated, so they start getting angry because the person doesn't understand what they are saying.’ Lack of awareness about autism, then, was seen to exacerbate the negative side of the condition.

Being autistic meant that a person had difficulties taking the point of view of other people. This expressed itself in many ways; for example, individuals would say things that would be deemed offensive. Violet gave a description of Sara, one of her autistic riders, who told others that they would look much better if they wore contact lenses rather than glasses, or that they should get a new haircut. According to Violet, Sara said what she meant without considering how this might feel to the other person: ‘She has no conception of what I might be feeling if she said that’. At first glance, the trainers thus largely accepted the widely held view that autistic people lack empathy.

At times, though, descriptions during interviews challenged the notion that a lack of empathy was a permanent autistic neurological state. Millie defined autism as ‘no empathy’, yet went on to suggest that when riding and interacting with the horses, the children ‘are actually feeling a bit of empathy’. Violet also suggested that a lack of empathy was not a permanent physical deficit. She recounted a child in one of her classes: ‘Toffee's trot was getting slightly fast and the child said ‘mind yourself Violet, Toffee's going to run you over!’ And now that again is a huge transference of thought onto someone else’.

We can gain some insight as to trainers’ ambiguous reports regarding riders’ capacity for empathy, and indeed how the therapy *works*, by looking at the aforementioned underexplored sensory aspects of the disorder. Centre staff frequently noted the sensory issues faced by riders. Heightened sensory awareness was widely reported by trainers at the Centre, echoing reports by both autistic individuals and their parents (Chamak et al. 2008).

For trainers and parents, sensory overload could strike even neurotypical people. During a training session run by Pauline, the mother of autistic boy Tom, Centre staff were given an insight into the daily difficulties of living with sensory issues. Pauline highlighted how easy it is for those who did not experience autism to underestimate the complexity of their environments. A regular supermarket posed no particular problem if appropriate ‘filtering’ is in place, but for her son it presented a severe sensory overload of glaring lights, continuous music, bakery smells, and noise from check out machines.

Pauline then asked the participants to remember a list of ten words whilst a strobe light flashed, heavy metal music played at top volume, and hairspray was sprayed in their faces. They found it impossible to remember any of the words given to them. Through the exercise, Pauline sought to re-create in people without autism the bewilderment of sensory overload, and demonstrate its effect on one's abilities to learn and to socialize. Pauline explained that what could not be reproduced, though, was the kind of ‘stimming’ her son Tom would exhibit to rebalance and cope with overload. These practices included flapping his hands, as well as banging objects together.

Echoing Pauline's understanding, trainers deemed difficulties in empathizing as symptoms of a fundamental problem of ‘filtering’ sensory input, rather than the result of a complete structural deficit. Jacqui, who had extensive experience in working with a range of people with learning disabilities and mental health disorders, was particularly clear on the fact that lack of empathy in autistic individuals was caused by ‘not being able to filter in the brain’. Insufficient filtering led to a cognitive ‘overload’, and it was issues in processing that caused the perceived lack of empathy: ‘Of course they don't have empathy. It's a filtering thing. They have so many things going on at the one time’. The physical environment in the riding arena is meant to be sensorially calm (Davidson 2010) to enhance the calmness of the horse. Flickering or harsh lights are not permitted, novel or distracting objects are banned.

Trainers at the Centre hence saw autistic symptoms – in particular, an absence of apparent empathy – not as permanent neurological deficits of an individual, but as an expression of a problem co-constituted by the social and spatial surroundings. The possibility existed that surroundings could be modulated so that overload was minimised and persons with autism could feel more at ease. This would help to maintain a level of calm, limiting responses such as stimming and instead providing an opportunity to be more open to the surroundings without getting overwhelmed.

Overall, we can see that the trainers interviewed detected many social and environmental facets of autism that add nuance to a generalized belief in a brain-based model of impairment. ‘Empathy’ loomed large in the discussions with respondents: the need for social institutions and individuals to be more empathetic towards individuals with autism was foregrounded, deficits in empathetic expression as symptoms (perhaps even the quintessence) of autism were reflected upon, and empathy as an ambivalent property or process came to be regarded as the target and evidence of therapeutic intervention. It is this latter aspect that we will consider in more detail in the remaining sections.

## Embodied interaction

As discussed above, respondents at the Centre highlighted that autistic riders showed more empathy during equine therapy sessions. It was commonly noted that the children were more likely to interact with the horse than the person leading their horse, or with the class instructor. Laura said, ‘*Initially*, a lot of the riders will communicate a lot more with the horses than they will with people’. Yet, ‘over time they start to talk to the leaders or the helpers… so they learn how to communicate’.

The embodied experience of riding the horse was a highly important factor for all of the respondents in their accounts of the therapeutic effects of equine interaction. As Jacqui exclaimed: ‘The horse is the tool! We can't do it [without the horse]. Because a horse you are sitting on … not all animals they can sit on and be individual and be independent’. For Laura, interacting with horses was ‘a matter of body language… [i]t's [vocal] tone, behaviour, their body language … [The children at the Centre] can just talk to [the horses], or use their legs, or give them the instruction whether it's using your voice or your body’. Indeed, such embodied interaction had educational effects beyond learning to ride; in Angela's words: ‘as in left and right, up and down, they pick that up at horse riding, as opposed to in school when it's confusing for them … because at the horse riding, they are actually doing the motion, it is not just the language, it's a body language thing as well’.

The ‘feeling’ of horse riding was deemed salient in discussions of the efficacy of equine therapy. For Angela, ‘it's the feeling the horse gives them once they are on top of that horse’. She went on to discuss the transformation of the child's attitude towards riding prior to and after the embodied experience of riding: ‘normally before we go we take the children and let them see the horse riding. Let them have a think about it first. And there's a different attitude at that stage from when they actually go on the horse and come off of it. So that changes’.

The role of each specific horse in shaping the rider-ridden interaction was noted by all the service providers at the Centre. Each horse was described as having its own ‘personality’ that was integral in facilitating this learning process. For example, Jacqui described some of the difference between the animals at the Centre: ‘Well look at the difference between Zack and Rolo … Zack is smaller and he's fast, and he doesn't like to stand still … Whereas Rolo is fat, lazy, slow and doesn't give a toss. You know, and that's his personality’.

All trainers noted the importance of bringing riders' individual needs in tune with individual horses' personalities. Violet reflected that ‘sometimes you do want a really safe seat, like Cody or DD, or Raffles’ whereas for other riders, more challenging horses work better: ‘Zara has just enough to keep [the rider] alert and on the edge of her saddle as it were’. Indeed, horses were chosen to become part of the team at the Centre particularly as a result of their personalities. It was suggested that a good range of horses from reliable ‘safe seats’ to unpredictable ‘quirky ponies’ were necessary to cater for the range of autistic riders and their sensory experience. Some more predictable horses were described as ‘push button rides’ and others as ‘exciting’ or ‘challenging’. The horse Zara, for example, was described by Diane as a good therapy horse since she was ‘safe and steady and predictable’. For those with sensory hypersensitivity, placid horses were used as ‘safe seats’ to ensure that the rider would be able to remain calm and focused. For hyposensitive riders in need of stimulation, a ‘quirky’ or unpredictable horse was chosen. Riders who were ‘hyperactive’ or ‘a bit cheeky’ also were given a more challenging ‘quirky’ horse or pony such as Hope or Zack to ride. Horses reflected their riders’ needs, whose embodied practices were in turn shaped in important ways by the nature of the equine interaction. The relation between rider and horse was continually reassessed. This ‘becoming with’ (Haraway 2003; 2006) was partial (Latimer 2013): delicately calibrated, formed, and reformed anew each week.

Despite noting the predictability of certain horses, Jacqui said, ‘All horses are unpredictable. You could have the safest, calmest of horses. But you can't stop a bird from jumping out of a bush at the wrong time and the horse gets a fright. Horses are flight animals … They are unpredictable, but then so are kids with autism’. Violet also referred to the importance of unpredictability in the horses’ behaviour in the efficacy of the therapy for some riders:

Violet: It depends on the rider, because if they are difficult, you don't want to put them on a horse like Zack, forward going. Whereas if they are in their own deep little world and nothing that you can say can snap them out of it, then I'm all for putting them on a Zack.

RM: So what does Zack do for you to make that choice?

Violet: Well you never know what he is going to do next. He's young, and slightly unpredictable.

This unpredictability of both horse and rider at times produced effects considered by parents, carers, teachers, and to some extent practitioners, as miraculous. As a result of equine therapy sessions, autistic children were seen as becoming more communicative and increasingly aware of self and others (be those humans or other animals). This proliferation of sociality was taken to be indicative of the positive therapeutic effects of horse riding. We can see too that properties unique not only to horses per se but to specific horses and indeed to their riders were regarded as integral to the efficacy of the therapy.

## Understanding efficacy

When asked about any significant experiences at the Centre, staff and parents often described ‘breakthrough’ moments, significant improvements in social functioning of riders as a result of weekly sessions at the Centre. Diane noted being surprised at times by children's ability to understand and communicate better: ‘surprisingly enough, you think they're not with you and they're not paying attention, and they end up doing something that's completely not what you were expecting, just to prove that they were listening all along’. Whilst discussing one of her students, teacher Angela said: ‘I'm quite surprised at how well he's come on. He's actually trotting…And that's amazing! I would never have said he would have got to where he is now’. These events largely centred on the emergence of language use and other forms of communication and empathic awareness. In particular, service providers noted the significance of the emergence of eye contact when describing sessions with autistic riders:

[It] builds up… and then over time you then get maybe the eye contact. It may be just an absolute split second: if you blink, you'll miss it. It might take months; it might take years but you get that little bit of interaction … One day I just looked up and [James] just looked down at me and smiled! And then looked away again. It was like my God, brilliant. (Laura)

Angela talked about how autistic children would sometimes start to talk while riding on the horse. The momentary experience of feeling the horse's movement and their own movement ‘just opens up their world’. The children were seen to ‘open up’ to the social world around them at the Centre by displaying normative communication behaviours. This ‘opening up’ was bidirectional. The emerging abilities of the child (widely conceived as *surprising* and novel progressions, arising out of the therapy's property of unpredictability) ‘opened up’ perceptions of the rider's abilities, and relatedly, the social world into which they emerged.

For Laura, horses communicated in ways that were attuned for autistic forms of interaction. Whilst emphasizing the role of eye contact in therapeutic progress, she noted that the horse ‘neve*r wants* eye contact’, avoiding ‘putting pressure onto the rider’. For Laura, human communication could be seen as being constrained by societal conventions: ‘it's a socially acceptable thing to look directly in somebody's face and ask them a question or give them an instruction … I think a lot of autistic kids relate to animals in general because they don't ask for that socially acceptable thing in return’.

While the effects of therapy were clear, for some respondents at least the mechanism of action – beyond ‘horse riding’ – remained opaque. Jacqui was in many ways uncertain about how equine therapy worked: ‘I have no reason for it. I have no idea. I don't know and I don't understand how they work. But what I do know is that the horses make some sort of difference … I don't know that you could [understand it]’. And, yet, when thinking about the ‘stimming’ behaviours (such as hand flapping or banging objects together) of Tom and the therapeutic effects of riding, she reflected:

Definitely it's the pace of movement … it makes them stable, just keeps them calm while at the same time stimulating them … A lot of it is to do with the rhythm of the horse, you know, a constant rhythm. Because that's what autistics like, same, same, same, same.

Themes of domination, submission, and liberation were also part of the narratives of Centre staff and parents. Violet suggested that autistic service users ‘have a certain power to control [the horse's] movement. I mean basically in most human beings there is a sort of power game, is there not?’ In effect, riding up high and controlling a strong, powerful animal was regarded as an empowering experience: when riding a horse, an autistic person is offered an all too infrequent opportunity to embody a position of power in a social context. When discussing the therapeutic efficacy of equine therapy, Jacqui reflected that autistic riders are given an opportunity of ‘feeling free’. In particular, this freedom related to being free from the physical control of other people:

They are not being pinned down. They are above everybody else…They are maybe used to people towering over them and if anything happens then they can be pinned. When they are on a horse they can't be pinned, because they are up above everybody else.

As she further suggested:

[Autistic riders] are giving the instructions to the horse, not receiving the instructions. And the horse will move, and the horse will take direction. So that is a line of communication as well, that they won't be used to having with human beings.

Angela likewise supported the importance of the rider gaining some kind of control, describing times when one boy took his horse back to the stables to untack after the class. She exclaimed:

the empowerment when he is walking back! They don't have many moments like that. You know, when he is so proud of himself … it's some experience! It really is. For them it's just totally amazing!

Yet, in spite of the positive experiences that a child at the Centre may have, they can be replaced by other children who were deemed to have a better chance of ‘progressing’. As Millie put it: ‘you know the whole point of going to horse riding is that they do progress’. An experience of pleasure while riding was not sufficient: ‘They might enjoy it but they also need to make progression’. ‘Progress’ involved a gradual reorientation of the autistic child, through equine interaction, to a more functional form of life – a complex choreography of normalization. The spectre of the lack of funding and support for autistic people in adulthood always loomed large at the Centre, and the need to do as much as possible to ameliorate symptoms whilst some kind of support was available was a powerful motor for action. As Millie suggested, once adulthood was reached the support of a school environment is removed. Only one dedicated day centre exists for the estimated 6,500 autistic adults living in the city where this study was based. With very limited access to funding for full time care, the need to normalise was deemed considerable. The Centre, then, had to be firmly fixed on riders’ progression, and removing children who were not making satisfactory progress ensured that limited resources were targeted towards those who might.

Whilst as we have seen, the mechanism (and for some, perhaps even the effects) of progression was not always clear, progress per se was considered possible. As Lucy said: ‘I haven't figured it out yet, what it brings to the autistic ones. But it brings something’. For practitioners, this ‘something’ seemed to relate to the dynamic juxtaposition or becoming with (Haraway 2003) of human and equine bodies. Particularly salient was the pairing with equine personalities: delicate calibration of safe seats or quirky ponies, with hyper- or hyposensitive riders, and the physical architecture of the calm space, were seen as key to alleviating processing issues faced by many of the children. The use of eye contact and the emergence of language were used by practitioners, parents, teachers, and carers as surprising and novel, and evidence of ‘progress’ in a child's social functioning. A reduction in the salience of issues around social interaction, communicative ability, and stereotypical behaviours (key matters for many working in formal mental health services) were evident in children who had participated in the activities of the Centre, and hence served as testament to the efficacy of equine therapy.

## Conclusion

A variety of social scientific work, from within anthropology and beyond, has considered autistic experience. This paper contributes to this growing body of scholarship through attention to one form of therapeutic intervention: equine therapy. In so doing, our analysis speaks to a wider move within the interdisciplinary field of animal studies to consider the origins, proliferation and practices of therapies wherein animal-human interactions are invoked as the quintessence of their modalities (Berget, Ekeberg, and Braastad 2008; Friedmann et al. 2013; Lanning et al. 2014; Nimer and Lundahl 2007; Odendaal 2000; O'Haire 2013; Ormerod et al. 2005; Solomon 2010; 2015, Sterba 2006).

In particular, this analysis has considered how staff and the parents of riders at a horse therapy centre account for the mechanisms and successes of equine therapy – success that is largely understood through and evidenced by processes of empathy. As Angela reflected, for children with autism, equine therapy ‘just opens up their world’. The horse, by helping to reorient young autistic riders to a more functional ‘form of life’ (Wittgenstein 1953), reoriented service providers, carers, and parents themselves towards a perception of a more textured inner world experienced by children with autism. It is in part through this perception, we suggest, that particular narratives of therapeutic efficacy come to make sense to Centre staff and parents. Overall, three key explanations emerged to explain the efficacy of equine therapy: first, the sensorial, embodied experience of riding the horse; second, the specific movements and rhythms of the horse; and, finally, the personality of the horse. Through the multisensory dynamics of therapy, autistic children came to ‘surprise’ parents and teachers with their intersubjective, communicative, and empathic abilities.

Such empathetic effects recast young autistic riders (and those who work with, and account for, these children) as agents participating in the generation of new(er) understandings of autism by contradicting common notions of the condition that figure autistic experience as necessarily removed from intersubjective relations. The horse was seen as inimitable in achieving these breakthroughs in communication. Initially described as a magical ability, the therapeutic efficacy of the horse was presented not simply as a function of the liveliness of animality per se but as a phenomenon unique to horses. Key horse-specific properties included communicative form and the ability to facilitate the embodied experience of riding, with its particular movement and rhythm. Indeed, these delineated further: the personality of particular horses (e.g. as a predictable ‘safe seat’ or a ‘quirky pony’) was understood to align with, uncover, and elaborate the personalities of specific riders. These properties and their efficacy were sometimes stable and at other times underpinned by an unpredictability that offered surprise (particularly for parents, carers, and practitioners).

Our respondents’ reflections on empathy – and the empathetic features intrinsic to this mode of consideration – prompt us to conclude with some brief notes on intersubjectivity (which we take to be a key enabler and product of empathy). Over time, discussions of intersubjectivity have often confined this concept to something achieved through the cognitive processes of humans and not animals, and especially through the use of language. Yet, such framings fail to fully resonate with the empathetic processes and their associated therapeutic effects promoted and reflected upon by respondents when discussing the rider-ridden relational assemblage of equine therapy. The multiple intersubjectivities encountered at the Centre – between humans and between species – were integral to perceptions of therapeutic efficacy. Indeed, any exclusion of the relational and the sensorial in discussions of equine therapy would render explanations of this incomplete. Accordingly, the accounts and experiences of the Centre parents and staff extend our understandings of empathy beyond the anthropocentric and hyper-discursive version of this concept that predominates in many western societies, and within the human sciences that have taken these as their objects of study. Humans and (other) animals intertwine frequently, customarily, and (un)predictably; an understanding of how this happens in practice, and with what effects, requires a revitalised attention to the empathetic processes enabling and further complicating these material-semiotic hybrids. In so doing, we are invited into new reflections on the ‘ontological choreography’ (Haraway 2003, 51) of lively interactions.

## Notes

1. In this paper, we use a variety of terms interchangeably to describe the clients of therapy, such as ‘autistic riders,’ ‘autistic person,’ or ‘person on the autistic spectrum.’ We are fully aware of the politics around different terms used to describe people diagnosed on the autism disorder spectrum, but we think that the argument developed here does not depend on specific labels, and instead deliberately use diverse terms that mirror the diversity of usage in the field-site and elsewhere.

2. For example, the film ‘Horse Boy™’ was released in 2009, documenting the journey of the parents of Rowan, a young autistic boy, to find a cure for their son (Horse Boy™ Foundation 2012). See also: ‘The Horse Boy™: Bringing equine therapy to help autistic children in Britain’ (Hunt, The Telegraph, 2009), ‘Horse power: How a pioneering therapy taught this autistic little boy how to love’ (Anon, The Daily Mail, 2010), ‘How riding a horse taught a boy to speak: A father has found a therapy that can transform autistic children’ (Roberts, The Independent, 2012).

3. No interviews with autistic children or adults were carried out. This is a recurring limitation in autism studies research that will be addressed in forthcoming research.
